# Neighborhood Disadvantage in a Nationally Representative Sample of Community-Living Older US Adults

**DOI:** 10.1001/jamanetworkopen.2024.50332

**Published:** 2024-12-12

**Authors:** Thomas M. Gill, Linda Leo-Summers, Brent Vander Wyk, Robert D. Becher, Jingchen Liang

**Affiliations:** 1 Department of Internal Medicine, Yale School of Medicine, New Haven, Connecticut; 2 Department of Surgery, Yale School of Medicine, New Haven, Connecticut; 3 Yale School of Public Health, New Haven, Connecticut

## Abstract

**Importance:**

Although neighborhood socioeconomic disadvantage has become the standard for evaluating contextual socioeconomic deprivation at the Census-block level, little is known about its prevalence or association with long-term mortality in nationally representative samples of older persons.

**Objectives:**

To estimate the prevalence of neighborhood disadvantage among a nationally representative sample of community-living older adults; to identify how prevalence estimates differ based on relevant demographic, socioeconomic, geographic, clinical, and geriatric characteristics; and to evaluate the association between neighborhood disadvantage and all-cause mortality.

**Design, Setting, and Participants:**

This cohort study analyzed linked data of community-living persons aged 65 years or older in the contiguous US participating in the National Health and Aging Trends Study (NHATS) from 2011 to 2021. Data on demographic, socioeconomic, geographic, clinical, and geriatric characteristics were obtained primarily from the baseline NHATS assessment. NHATS survey weights were used for all analyses. Data analysis was conducted from February to July 2024.

**Main Outcomes and Measures:**

Neighborhood disadvantage—the main measure for each of the 3 objectives—was assessed using the area deprivation index, which was dichotomized at the worst quintile (defined as the worst 2 deciles). Ascertainment of mortality over 10 years was 100% complete.

**Results:**

Among the 7505 participants, with a weighted mean (SD) age of 75.3 (7.4) years, 56.8% were female, 6.6% were Hispanic, 8.2% were non-Hispanic Black, and 81.7% were non-Hispanic White individuals. The prevalence of neighborhood disadvantage was 15.8% (95% CI, 14.9%-16.7%), but it differed greatly across multiple subgroups. The largest differences after adjustment for age and sex were observed for non-Hispanic Black compared with non-Hispanic White participants (rate ratio [RR], 3.11; 95% CI, 2.56-3.79); those with less than a high school diploma vs college degree or higher educational level (RR, 3.47; 95% CI, 2.75-4.39); and those in several Census divisions, with an RR as high as 7.31 (95% CI, 2.98-17.90) for West South Central vs Pacific. The mortality rates were 48.5% (95% CI, 44.6%-52.1%) and 43.5% (95% CI, 42.2%-44.7%) among participants in a disadvantaged and a nondisadvantaged neighborhood. Neighborhood disadvantage was associated with mortality after adjustment for demographic characteristics (hazard ratio [HR], 1.25; 95% CI, 1.11-1.40) but not after further adjustment for socioeconomic characteristics (HR, 1.11; 95% CI, 0.98-1.25).

**Conclusions and Relevance:**

In this cohort study of community-living older adults, population-based estimates of neighborhood disadvantage differed greatly across multiple subgroups. This contextual indicator of socioeconomic deprivation was associated with long-term mortality, but the association was diminished and no longer significant after accounting for individual-level socioeconomic characteristics.

## Key Points

**Question:**

How does the prevalence of neighborhood socioeconomic disadvantage differ according to key characteristics of older persons and is neighborhood disadvantage associated with all-cause mortality?

**Findings:**

In this cohort study of 7505 participants in the National Health and Aging Trends Study, the prevalence of neighborhood disadvantage was 15.8% but differed greatly across multiple subgroups, with the largest differences observed for race and ethnicity, educational level, and US Census division. Neighborhood disadvantage was associated with 10-year mortality but not after adjustment for participant socioeconomic characteristics.

**Meaning:**

Findings of this study suggest that substantial heterogeneity exists in the prevalence of neighborhood disadvantage; the association between this contextual indicator of socioeconomic deprivation and long-term mortality may largely be attributable to individual-level socioeconomic characteristics.

## Teaser

This cohort study explores the prevalence of neighborhood disadvantage and its association with individual characteristics and mortality among Medicare beneficiaries aged 65 years or older.

## Introduction

Neighborhood disadvantage is a well-established social determinant of health and functional well-being.^1^ A neighborhood may be disadvantaged based on indicators of education, employment, housing quality, and poverty. Prior research has shown that living in a socioeconomically disadvantaged neighborhood confers increased risk for several adverse outcomes, including mortality,^2^ hospital readmission,^3^ incidence and severity of delirium after major surgery,^4^ Alzheimer disease neuropathology,^5^ and reduced active life expectancy,^6^ among others.

Since the development and release of the Neighborhood Atlas in 2018,^1^ hundreds of articles have been published on neighborhood disadvantage at the US Census-block level using the area deprivation index (ADI). Prior studies had largely evaluated area deprivation at less granular geographic units such as county^7^ or Census tract,^8^ which are too large and heterogeneous to represent discrete neighborhoods.^9^ Despite the burgeoning literature on the ADI at the Census-block level, little is known about the prevalence of neighborhood disadvantage in nationally representative samples of older adults in the US. In 2023, the National Health and Aging Trends Study (NHATS), a prospective, population-based longitudinal study of Medicare beneficiaries aged 65 years or older,^10^ made block group files available under limited circumstances to facilitate linkage to contextual data, such as the ADI.

Using these linked data from NHATS, we set out to accomplish 3 objectives: (1) to estimate the prevalence of neighborhood disadvantage among a nationally representative sample of community-living older adults; (2) to identify how these prevalence estimates differ based on relevant demographic, socioeconomic, geographic, clinical, and geriatric characteristics; and (3) to evaluate the association between neighborhood disadvantage and all-cause mortality. The results of this study are intended to advance the understanding of neighborhood disadvantage, a granular contextual indicator of socioeconomic deprivation, at the national level and establish a strong foundation for future population-based research on health disparities.

## Methods

### Study Population

On September 30, 2010, NHATS drew a random sample of persons aged 65 years or older living in the contiguous US (excluding Alaska, Hawaii, and Puerto Rico) from the Medicare enrollment file. Counties were sampled from regional strata, and non-Hispanic Black persons and those aged 90 years or older were oversampled within zip codes. Baseline (round 1) assessments, completed from May through November 2011, yielded a sample of 8245 persons with a 71% weighted response rate. Proxy respondents were interviewed when the participant could not respond (n = 583, or 5.8% weighted). This cohort has been linked to Medicare and geographic data.^10^ These restricted NHATS data are available through a Data Use Agreement, which requires an approved application and data protection plan. The Johns Hopkins University Institutional Review Board approved the NHATS protocol, and all NHATS participants provided written informed consent. The Yale University Institutional Review Board approved the current cohort study and its use of NHATS data. We followed the Strengthening the Reporting of Observational Studies in Epidemiology (STROBE) reporting guideline.^11^

### Data Collection

During round 1 of NHATS, information was collected on demographic, socioeconomic, geographic, clinical, and geriatric characteristics.^10,12^ The demographic characteristics included age, sex, and self-reported race and ethnicity. Race and ethnicity (ie, Hispanic, non-Hispanic Black [hereafter Black], non-Hispanic White [hereafter White], and other [Asian, American Indian or Alaska Native, Native Hawaiian or Pacific Islander, other, do not know, or more than 1 race and ethnicity]) data were analyzed for descriptive purposes. The socioeconomic characteristics included educational level, annual income, and Medicaid eligibility, which was obtained from the Medicare records. Annual income for the participant (and partner, if applicable) was assessed using a composite from Social Security benefits; Veterans Affairs benefits; pension plans; retirement plans; mutual funds, stocks, and bonds; and checking and savings accounts.^13^ Missing data on income were imputed using values provided by NHATS.^12^ An annual income less than $15 000 was classified as low.^14,15^

The geographic characteristics included Census division (eFigure in Supplement 1) and rural residence based on a 2013 Rural-Urban Continuum Code of 4 to 9.^16^ The clinical characteristics included 9 self-reported, physician-diagnosed chronic conditions: heart attack, high blood pressure, arthritis, osteoporosis, diabetes, lung disease, stroke, cancer, and hip fracture since age 50 years. The geriatric characteristics included frailty and dementia. Participants were categorized as nonfrail, prefrail, and frail (according to the Fried phenotype^17^) and as having no dementia, possible dementia, or probable dementia (based on a validated assessment strategy developed by NHATS^18,19^).

Neighborhood disadvantage was assessed with the ADI, a Census-based socioeconomic index.^1,3^ As shown in the eTable in Supplement 1, the ADI includes 17 educational, employment, housing quality, and poverty indicators obtained from the American Community Survey (ACS). These indicators are weighted and summed to yield a score for each neighborhood at the Census-block level.^7^ For the current study, we used the Neighborhood Atlas to link block-group data from NHATS to the 2013 ADI file, representing ACS data from 2009 to 2013.^3^ The ADI national percentiles were categorized into national deciles, with higher decile scores indicating greater socioeconomic disadvantage. Based on its established threshold effects,^3^ these scores were dichotomized at the eighth decile to distinguish the disadvantaged group (9-10 deciles) from the nondisadvantaged groups (1-8 deciles). Because of privacy concerns, NHATS does not permit analysis or reporting of ADI scores as percentiles.^20^

### Analytic Sample and Outcome

Among the 7609 NHATS participants who were living in settings other than nursing homes (ie, community living) at the time of their round 1 assessment, 104 (1.4%) lacked ADI scores, leaving 7505 participants in the analytic sample. ADI scores were not available for 2 reasons: missing values in the source ACS data (n = 86) and high group quarters population or low population and/or housing (n = 18), as explained elsewhere.^21^

For the longitudinal analysis, we evaluated mortality rates over the 10-year follow-up and time to death within 10 years. Deaths were ascertained from the Medicare enrollment files.^22^

### Statistical Analysis

NHATS survey weights were used for all analyses,^23^ thereby permitting national estimates. Descriptive statistics were calculated for participants in the analytic sample but could not be compared with those of the 104 excluded participants because of small, unweighted cell sizes.^24^ Descriptive characteristics were also calculated for the ADI decile scores, including mean (SD), median (IQR), number (%) for each decile, and number (%) for the worst quintile, denoting neighborhood disadvantage. The prevalence of neighborhood disadvantage was calculated within subgroups defined on the basis of the demographic, socioeconomic, geographic, clinical, and geriatric characteristics. Adjusted risk ratios (with 95% CIs) were subsequently calculated. The values for age were adjusted for sex, the value for sex was adjusted for age, and all other values were adjusted for age and sex. For consistency and to facilitate interpretation, comparisons for each characteristic were generally made to the group with the lowest prevalence. The corresponding *P* values were adjusted for multiple comparisons using the Benjamini and Hochberg procedure.^25^

Ten-year mortality rates were calculated, and cumulative mortality curves were generated^26,27^ according to the presence of neighborhood disadvantage. The independent association between neighborhood disadvantage and mortality was evaluated using a series of hierarchical Cox proportional hazards regression models that sequentially adjusted for the demographic, socioeconomic, geographic, clinical, and geriatric characteristics. Robust SEs and their corresponding 95% CIs were calculated by fitting each Cox proportional hazards regression model using the balanced repeated replication approach, as previously recommended.^28,29^ This approach accounts for the complex survey design,^23^ and it overcomes potential bias in estimated variance when proportional hazards assumptions are violated.^30^

All analyses were performed using SAS, version 9.4 (SAS Institute Inc) and R, version 4.4.0 (R Core Team). Statistical significance was defined as a 2-tailed *P* < .05. Data analysis was conducted from February to July 2024.

## Results

As shown in Table 1, the weighted mean (SD) age of the 7505 NHATS participants in the analytic sample was 75.3 (7.4) years. Among these participants, 56.8% were females and 43.2% were males, of whom 8.2% self-identified as Black, 6.6% as Hispanic, 81.7% as White, and 3.5% as other race and ethnicity. Altogether, 71.2% did not complete college, 21.0% had an annual income less than $15 000, and 13.4% were Medicaid eligible. The most common and least common Census divisions were South Atlantic (19.5%) and Mountain (2.7%), respectively. A total of 18.1% of participants were rural residents, 19.9% had more than 4 chronic conditions, 13.8% were categorized as frail, and 10.0% had probable dementia.

**Table 1.** Baseline Characteristics of the Analytic Sample^a^

Characteristic	Participants, %	
	Unweighted	Weighted
No. of participants	7505	34 742 661
Demographic		
Age, mean (SD), y	77.7 (7.9)	75.3 (7.4)
Age group, y		
65-69	18.5	27.9
70-74	20.7	24.8
75-79	19.9	19.1
80-84	19.8	14.7
85-89	12.6	9.2
≥90	8.6	4.3
Sex		
Female	58.5	56.8
Male	41.5	43.2
Race and ethnicity^b^		
Hispanic	5.9	6.6
Non-Hispanic Black	22.1	8.2
Non-Hispanic White	69.1	81.7
Other^c^	2.9	3.5
Socioeconomic		
Educational level		
<High school	27.2	21.8
High school diploma or GED	27.7	27.8
>High school diploma	20.0	21.6
≥College degree	25.1	28.9
Annual income, $		
Median (IQR)	26 903 (14 376-50 000)	31 992 (16 732-59 865)
<15 000	26.1	21.0
Medicaid eligible	16.5	13.4
Geographic		
US Census division		
New England	4.8	5.9
Middle Atlantic	13.6	13.4
South Atlantic	20.8	19.5
East North Central	13.8	13.7
East South Central	7.1	6.4
West North Central	9.6	9.4
West South Central	10.8	10.9
Mountain	2.5	2.7
Pacific	16.9	18.1
Rural residence	18.5	18.1
Clinical		
Heart attack	15.4	14.1
High blood pressure	67.4	64.1
Arthritis	56.0	53.8
Osteoporosis	20.5	21.2
Stroke	11.8	10.0
Diabetes	25.3	23.9
Lung disease	15.2	15.4
Cancer	25.7	25.8
Hip fracture since age 50 y	5.0	4.1
No. of chronic conditions		
0	8.8	9.9
1	18.9	20.6
2	26.9	26.9
3	23.9	22.7
≥4	21.6	19.9
Geriatric		
Frailty phenotype		
Nonfrail	34.5	39.9
Prefrail	48.3	46.3
Frail	17.2	13.8
Dementia status		
No dementia	73.3	79.1
Possible dementia	13.1	10.9
Probable dementia	13.6	10.0

Abbreviation: GED, General Educational Development.

^a^ All values were based on National Health and Aging Trends Study (NHATS) survey weights except for the unweighted numbers. A total of 104 participants were excluded from the analytic sample because scores for the area deprivation index could not be obtained.

^b^ Self-reported by NHATS participants.

^c^ Includes those who self-identified as Asian, American Indian or Alaska Native, Native Hawaiian or Pacific Islander, other, do not know, or more than 1 race and ethnicity.

**Figure 1.**
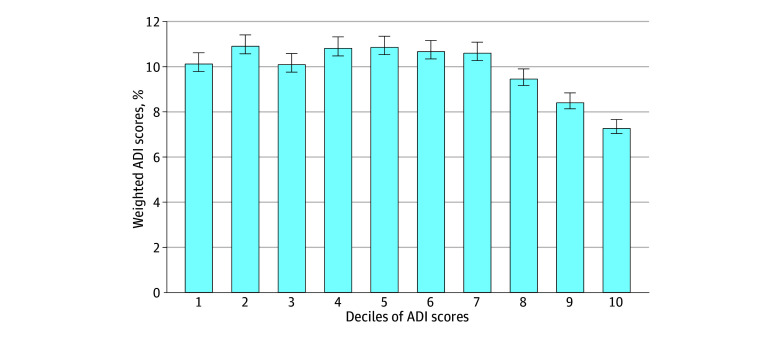
Distribution of Weighted Area Deprivation Index (ADI) Scores by Decile Among Community-Living Persons in the 2011 National Health and Aging Trends Study Cohort The weighted percentages are accompanied by SE bars.

The mean (SD) and median (IQR) ADI decile scores in the analytic sample were 5.3 (2.8) and 5 (2-7), respectively. The distribution of ADI scores by decile is shown in Figure 1. Scores in the first 7 deciles were overrepresented, while those in the last 3 deciles were underrepresented. The prevalence of neighborhood disadvantage, defined as the worst 2 deciles (or quintile), was 15.8% (95% CI, 14.9%-16.7%).

Table 2 shows the prevalence rates of neighborhood disadvantage according to relevant demographic, socioeconomic, geographic, clinical, and geriatric characteristics. Prevalence ranged from 4.7% for participants in the Pacific Census division to 41.0% for Black participants. Within subgroups, large absolute differences in prevalence (ie, exceeding 10%) were observed for race and ethnicity (from 7.4% for other to 41.0% for Black), educational level (from 8.2% or college degree and higher to 27.3% for less than high school), annual income (from 13.0% for $15 000 and higher to 26.4% for less than $15 000), Medicaid (from 13.6% for not eligible to 29.9% for eligible), Census division (from 4.7% for Pacific to 34.5% for West South Central), and residence (from 12.7% for nonrural to 30.1% for rural). In relative terms, the largest subgroup differences, adjusted for age and sex, were observed for Black compared with White participants (rate ratio [RR], 3.11; 95% CI, 2.56-3.79), less than a high school diploma vs college degree and higher educational level (RR, 3.47; 95% CI, 2.75-4.39), and West South Central vs Pacific Census divisions (RR, 7.31; 95% CI, 2.98-17.90). Differences by age group were modest, with the only significant difference observed for age groups 75 to 79 years vs 90 years or older (sex-adjusted RR, 1.34; 95% CI, 1.05-1.72).

**Table 2.** Prevalence of Neighborhood Disadvantage by Relevant Demographic, Socioeconomic, Geographic, Clinical, and Geriatric Characteristics^a^

Characteristic	Prevalence, %	Adjusted RR (95% CI)^b^	*P* value^c^
Demographic			
Age group, y			
65-69	16.8 (14.8-18.8)	1.33 (1.00-1.75)	.06
70-74	14.7 (12.9-16.5)	1.16 (0.90-1.50)	.28
75-79	17.1 (15.2-19.0)	1.34 (1.05-1.72)	.03
80-84	15.2 (13.4-17.0)	1.19 (0.93-1.52)	.17
85-89	15.5 (13.1-17.8)	1.21 (0.96-1.52)	.13
≥90	12.9 (10.2-15.6)	1 [Reference]	NA
Sex			
Female	16.4 (15.3-17.6)	1.10 (0.99-1.23)	.11
Male	15.0 (13.7-16.4)	1 [Reference]	NA
Race and ethnicity^d^			
Hispanic	21.5 (17.3-25.6)	1.62 (1.07-2.47)	.04
Non-Hispanic Black	41.0 (38.4-43.6)	3.11 (2.56-3.79)	<.001
Non-Hispanic White	13.2 (12.2-14.1)	1 [Reference]	NA
Other^e^	7.4 (3.5-11.2)	0.55 (0.26-1.19)	.15
Socioeconomic			
Educational level			
<High school diploma	27.3 (25.0-29.5)	3.47 (2.75-4.39)	<.001
High school diploma or GED	15.9 (14.3-17.6)	1.98 (1.57-2.49)	<.001
>High school diploma	14.4 (12.4-16.3)	1.77 (1.37-2.28)	<.001
≥College degree	8.2 (6.9-9.5)	1 [Reference]	NA
Annual income, $			
<15 000	26.4 (24.1-28.6)	2.07 (1.82-2.36)	<.001
≥15 000	13.0 (12.1-14.0)	1 [Reference]	NA
Medicaid eligibility			
Yes	29.9 (26.9-32.9)	2.21 (1.92-2.54)	<.001
No	13.6 (12.7-14.5)	1 [Reference]	NA
Geographic			
US Census division			
Middle Atlantic and New England^f^	7.3 (5.9-8.7)	1.55 (0.51-4.76)	.47
South Atlantic	19.3 (17.1-21.5)	4.09 (1.73-9.70)	.004
East North Central	16.7 (14.2-19.1)	3.57 (1.52-8.38)	.008
East South Central	26.8 (22.6-31.0)	5.70 (2.06-15.80)	.003
West North Central	20.4 (17.3-23.4)	4.34 (1.29-14.60)	.03
West South Central	34.5 (30.9-38.1)	7.31 (2.98-17.90)	<.001
Mountain	5.1 (1.9-8.4)	1.08 (0.39-3.03)	.90
Pacific	4.7 (3.3-6.0)	1 [Reference]	NA
Rural residence			
Yes	30.1 (27.5-32.7)	2.39 (1.35-4.21)	.006
No	12.7 (11.8-13.6)	1 [Reference]	NA
Clinical	30.1 (27.5-32.7)		
Specific chronic conditions			
Heart attack			
Yes	20.2 (17.6-22.7)	1.38 (1.17-1.62)	<.001
No	15.1 (14.2-16.1)	1 [Reference]	NA
High blood pressure			
Yes	17.4 (16.3-18.6)	1.35 (1.17-1.55)	<.001
No	13.0 (11.6-14.4)	1 [Reference]	NA
Arthritis			
Yes	17.4 (16.2-18.6)	1.24 (1.10-1.40)	.002
No	14.0 (12.7-15.2)	1 [Reference]	NA
Osteoporosis			
Yes	16.4 (14.4-18.4)	1.01 (0.86-1.18)	.95
No	15.7 (14.7-16.7)	1 [Reference]	NA
Stroke			
Yes	20.6 (17.6-23.6)	1.38 (1.22-1.57)	<.001
No	15.3 (14.4-16.2)	1 [Reference]	NA
Diabetes			
Yes	20.2 (18.2-22.2)	1.41 (1.25-1.59)	<.001
No	14.4 (13.5-15.4)	1 [Reference]	NA
Lung disease			
Yes	19.8 (17.3-22.4)	1.30 (1.12-1.52)	.003
No	15.1 (14.2-16.0)	1 [Reference]	NA
Cancer			
Yes	14.1 (12.5-15.8)	0.87 (0.77-1.00)	.06
No	16.4 (15.3-17.4)	1 [Reference]	NA
Hip fracture since age 50 y			
Yes	18.1 (13.7-22.5)	1.18 (0.96-1.46)	.14
No	15.7 (14.8-16.6)	1 [Reference]	NA
No. of chronic conditions			
0	12.4 (9.8-15.0)	1 [Reference]	NA
1	12.0 (10.2-13.7)	0.97 (0.75-1.26)	.87
2	16.5 (14.8-18.3)	1.35 (1.04-1.74)	.04
3	15.8 (14.0-17.6)	1.30 (1.04-1.63)	.04
≥4	20.6 (18.4-22.8)	1.70 (1.33-2.18)	<.001
Geriatric			
Frailty phenotype			
Nonfrail	12.6 (11.3-14.0)	1 [Reference]	NA
Prefrail	16.9 (15.6-18.3)	1.38 (1.19-1.59)	<.001
Frail	21.4 (18.9-23.9)	1.81 (1.50-2.17)	<.001
Dementia status			
No dementia	14.8 (13.8-15.8)	1 [Reference]	NA
Possible dementia	19.4 (16.8-22.1)	1.38 (1.13-1.69)	.004
Probable dementia	19.7 (17.0-22.3)	1.44 (1.20-1.72)	<.001

Abbreviations: GED, General Educational Development; NA, not applicable; RR, rate ratio.

^a^ All values were based on National Health and Aging Trends Study (NHATS) survey weights.

^b^ The values for age were adjusted for sex, the value for sex was adjusted for age, and all other values were adjusted for age and sex. For consistency and to facilitate interpretation, the reference group was generally the subgroup with the lowest prevalence.

^c^ All values were corrected for multiple comparisons, with *P* < .05 denoting statistical significance.

^d^ Self-reported by NHATS participants.

^e^ Includes those who self-identified as Asian, American Indian or Alaska Native, Native Hawaiian or Pacific Islander, other, do not know, or more than 1 race and ethnicity.

^f^ New England had to be combined with Middle Atlantic because of its small unweighted cell size.^24^

Many of the chronic conditions were associated with neighborhood disadvantage, including heart attack, high blood pressure, arthritis, stroke, diabetes, and lung disease, with age- and sex-adjusted RRs ranging from 1.24 (95% CI, 1.10-1.40) for arthritis to 1.41 (95% CI, 1.25-1.59) for diabetes. Significant differences were also observed for 2 (RR, 1.35; 95% CI, 1.04-1.74), 3 (RR, 1.30; 95% CI, 1.04-1.63), and 4 or more (RR, 1.70; 95% CI, 1.33-2.18) chronic conditions vs 0. For the geriatric characteristics, prefrail and frail vs nonfrail as well as possible dementia and probable dementia vs no dementia were all associated with neighborhood disadvantage, with age- and sex-adjusted RRs of 1.81 (95% CI, 1.50-2.17) for the frail phenotype and 1.44 (95% CI, 1.20-1.72) for probable dementia.

**Figure 2.**
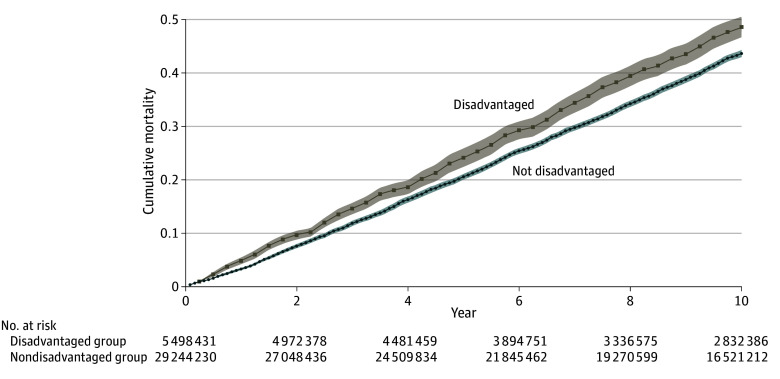
Cumulative Mortality Over 10 Years by Neighborhood Disadvantage National Health and Aging Trends Study–weighted values are shown, accompanied by SE bands. Fewer values are provided for the disadvantaged group because of small unweighted cell sizes.^24^ For all participants, the median (IQR) time to death was 5 (3-8) years.

Over the 10-year follow-up period, there were 3980 deaths, representing 15 389 064 survey-weighted deaths and 44.3% (95% CI, 43.0%-45.6%) mortality. The mortality rates were 48.5% (95% CI, 44.6%-52.1%) and 43.5% (95% CI, 42.2%-44.7%) among participants in a disadvantaged and nondisadvantaged neighborhood, respectively. Cumulative mortality for these 2 groups is shown in Figure 2. In a series of hierarchical Cox proportional hazards regression models, shown in Table 3, neighborhood disadvantage was associated with mortality over 10 years in the unadjusted analysis (hazard ratio [HR], 1.18; 95% CI, 1.05-1.33) and after adjustment for the demographic characteristics (HR, 1.25; 95% CI, 1.11-1.40). This finding was no longer statistically significant after adjustment for the socioeconomic characteristics (HR, 1.11; 95% CI, 0.98-1.25) but did not change much after additional adjustment for the geographic, clinical, and geriatric characteristics, respectively.

**Table 3.** Associations Between Neighborhood Disadvantage and Mortality Over 10 Years

Model No.	Covariates^a^	Hazard ratio (95% CI)^b^	*P* value^c^
1	Unadjusted	1.18 (1.05-1.33)	.008
2	Demographic characteristics	1.25 (1.11-1.40)	<.001
3	Demographic and socioeconomic characteristics	1.11 (0.98-1.25)	.09
4	Demographic, socioeconomic, and geographic characteristics	1.07 (0.95-1.21)	.26
5	Demographic, socioeconomic, geographic, and clinical characteristics	1.04 (0.92-1.16)	.56
6	Demographic, socioeconomic, geographic, clinical, and geriatric characteristics	1.06 (0.94-1.19)	.32

^a^ Demographic characteristics included age, sex, and self-reported race and ethnicity. Socioeconomic characteristics included educational level, annual income, and Medicaid eligibility. Geographic characteristics included US Census division and rural residence. Clinical characteristics included number of physician-diagnosed chronic conditions. Geriatric characteristics included frailty and dementia.

^b^ Values were calculated in a series of hierarchical Cox proportional hazards regression models that sequentially adjusted for the demographic, socioeconomic, geographic, clinical, and geriatric characteristics.

^c^ *P* < .05 indicated statistical significance.

## Discussion

In this nationally representative sample of community-living Medicare beneficiaries aged 65 years or older, we found that the prevalence of neighborhood disadvantage at the Census-block level was 15.8%. The prevalence differed significantly, however, according to many demographic, socioeconomic, geographic, clinical, and geriatric characteristics, with the largest absolute and relative differences observed for subgroups defined on the basis of race and ethnicity, educational level, and Census division, although substantive differences were also observed according to annual income, rural residence, several individual and multiple chronic conditions, frailty, and dementia. Living in a disadvantaged neighborhood was associated with an 18% increase in mortality over 10 years. This elevation in mortality risk was modestly increased after adjustment for the demographic characteristics but was considerably decreased and became nonsignificant after adjustment for socioeconomic characteristics, such as educational level, annual income, and Medicaid eligibility. The results from this population-based study demonstrate substantial heterogeneity in the prevalence of neighborhood disadvantage across multiple important subgroups of older adults in the US and suggest that the association between neighborhood disadvantage and long-term mortality may be attributable in large part to individual-level socioeconomic characteristics.

Population-based national estimates of neighborhood disadvantage are relatively sparse, with prior studies focusing on specific regions of the country^31^ or patient groups, such as veterans.^32^ To our knowledge, none has focused on community-living older persons or evaluated how neighborhood disadvantage differs according to relevant subgroups. Inherently, the prevalence of neighborhood disadvantage depends on the threshold used to dichotomize scores on the ADI. Although we chose the worst quintile, which has been commonly used in other studies,^4,5,6^ the prevalence of neighborhood disadvantage in the current study was only 15.8%. The lower-than-expected prevalence in this nationally representative sample could be due, at least in part, to the stratified multilevel sampling strategy of NHATS, which was based on select demographic and geographic factors.^33^

Although neighborhood disadvantage is a distinct social determinant of health, it mapped closely to participants’ race and ethnicity and to each of the individual-level socioeconomic characteristics, including educational level, income, and Medicaid eligibility. For example, 27.3% of participants with less than a high school education lived in a disadvantaged neighborhood vs only 8.2% of those with a college degree, leading to an adjusted RR of 3.47. These disparities for living in a disadvantaged neighborhood were also large for participants who had low annual income vs those who did not (26.4% vs 13.0%) and participants who were Medicaid eligible vs those who were not (29.9% vs 13.7%), leading to adjusted RRs that exceeded 2.0 for each characteristic. These results indicate that many older persons are disadvantaged socioeconomically at both the contextual and individual levels. The prevalence of neighborhood disadvantage was also substantially higher in rural than in nonrural communities and differed greatly by Census division in the contiguous US, with the highest rates observed in the West South Central and East South Central divisions and lowest rates observed in the Pacific and Mountain divisions. An earlier report showed similar results graphically by ADI deciles.^1^

Clinically, participants were more likely to live in a disadvantaged neighborhood if they had multimorbidity, defined as 2 or more chronic conditions, or if they had specific chronic conditions, such as heart attack, high blood pressure, stroke, diabetes, and lung disease, that disproportionately affect older persons with low socioeconomic status. Similar results were observed among participants who had frailty or dementia, 2 common geriatric conditions. Although temporal precedence cannot be established given the cross-sectional nature of these associations, the vulnerability to adverse outcomes among older persons living in disadvantaged neighborhoods may be further heightened when these clinical and geriatric risk factors are present.

Living in a disadvantaged neighborhood was associated with an increased risk of death over the course of 10 years, even after adjustment for age, sex, and race and ethnicity but not after further adjustment for the socioeconomic, geographic, clinical, and geriatric characteristics. The results of the hierarchical models suggest that adjustment for the socioeconomic characteristics had the largest role in the reduction of mortality risk. While prior nationally representative studies have demonstrated associations between area deprivation at the county level and 1-year mortality,^7^ to our knowledge, no study has evaluated the association between neighborhood disadvantage at the Census-block level and long-term mortality or has attempted to ascertain whether such associations persist after serial adjustment for demographic, socioeconomic, geographic, clinical, and geriatric characteristics. As the smallest available geographic unit of analysis, typically ranging from 600 to 3000 persons, Census-block groups likely capture individual-level exposure to neighborhood disadvantage more precisely than larger Census units.^9^ These smaller geographic units may be more amenable to targeted interventions designed to address deficiencies in resource-poor environments.^34^

Two additional strengths enhance the generalizability, validity, and applicability of these findings. First, by linking ADI data from the Neighborhood Atlas to participants of a well-phenotyped, population-based cohort from the NHATS, we were able to generate nationally representative estimates of neighborhood disadvantage in numerous subgroups of community-living older adults across multiple domains. This wealth of data is not available in nationally representative administrative datasets, such as those from the Centers for Medicare & Medicaid Services. Second, the ascertainment of neighborhood disadvantage was nearly 100% complete, and the ascertainment of death over 10 years was 100% complete.

### Limitations

This study has limitations. First, the results may not be applicable to the 4% of US adults aged 65 years or older who did not have Medicare in 2011 or to Medicare recipients outside of the contiguous US.^10^ Second, because NHATS does not permit analysis or reporting of ADI scores as percentiles, we were unable to evaluate whether or how the results might differ based on thresholds other than worst ADI quintile for identifying disadvantaged neighborhoods. The 2 most common alternative thresholds—worst 15th percentile and worst quartile—would lead to lower and higher prevalence estimates, respectively, but may not have substantively affected the subgroup differences or mortality results. Third, it was not possible to report the prevalence of neighborhood disadvantage (and associated RR) separately for the New England Census division because of its small unweighted cell size.^24^

## Conclusions

In this cohort study, the population-based estimates of neighborhood disadvantage differed greatly across multiple subgroups of community-living older adults in the US. This contextual indicator of socioeconomic deprivation was associated with long-term mortality, but the finding was no longer significant after accounting for individual-level socioeconomic characteristics.

## Notes

Supplement 1. **eFigure.** Census Divisions in the Contiguous United States **eTable.** Indicators of the Area Deprivation Index at the Census-Block Level

Supplement 2.
Data Sharing Statement

## References

[1] Kind AJH, Buckingham WR. Making neighborhood-disadvantage metrics accessible: the Neighborhood Atlas. N Engl J Med. 2018;378(26):2456-2458. doi:10.1056/NEJMp1802313 29949490 PMC6051533

[2] Hermes Z, Joynt Maddox KE, Yeh RW, Zhao Y, Shen C, Wadhera RK. Neighborhood socioeconomic disadvantage and mortality among Medicare beneficiaries hospitalized for acute myocardial infarction, heart failure, and pneumonia. J Gen Intern Med. 2022;37(8):1894-1901. doi:10.1007/s11606-021-07090-z 34505979 PMC9198133

[3] Kind AJ, Jencks S, Brock J, . Neighborhood socioeconomic disadvantage and 30-day rehospitalization: a retrospective cohort study. Ann Intern Med. 2014;161(11):765-774. doi:10.7326/M13-2946 25437404 PMC4251560

[4] Arias F, Chen F, Fong TG, . Neighborhood-level social disadvantage and risk of delirium following major surgery. J Am Geriatr Soc. 2020;68(12):2863-2871. doi:10.1111/jgs.16782 32865254 PMC7744425

[5] Powell WR, Buckingham WR, Larson JL, . Association of neighborhood-level disadvantage with Alzheimer disease neuropathology. JAMA Netw Open. 2020;3(6):e207559. doi:10.1001/jamanetworkopen.2020.7559 32525547 PMC7290421

[6] Gill TM, Zang EX, Murphy TE, . Association between neighborhood disadvantage and functional well-being in community-living older persons. JAMA Intern Med. 2021;181(10):1297-1304. doi:10.1001/jamainternmed.2021.4260 34424276 PMC8383163

[7] Singh GK. Area deprivation and widening inequalities in US mortality, 1969-1998. Am J Public Health. 2003;93(7):1137-1143. doi:10.2105/ajph.93.7.1137 12835199 PMC1447923

[8] Flanagan BE, Gregory EW, Hallisey EJ, Heitgerd JL, Lewis B. A social vulnerability index for disaster management. J Homel Secur Emerg Manag. 2011;8:23.

[9] Lou S, Giorgi S, Liu T, Eichstaedt JC, Curtis B. Measuring disadvantage: a systematic comparison of United States small-area disadvantage indices. Health Place. 2023;80:102997. doi:10.1016/j.healthplace.2023.102997 36867991 PMC10038931

[10] Freedman VA, Kasper JD. Cohort profile: the National Health and Aging Trends Study (NHATS). Int J Epidemiol. 2019;48(4):1044-1045g. doi:10.1093/ije/dyz109 31237935 PMC6934030

[11] von Elm E, Altman DG, Egger M, Pocock SJ, Gøtzsche PC, Vandenbroucke JP; STROBE Initiative. The Strengthening the Reporting of Observational Studies in Epidemiology (STROBE) statement: guidelines for reporting observational studies. Ann Intern Med. 2007;147(8):573-577. doi:10.7326/0003-4819-147-8-200710160-00010 17938396

[12] Kasper JD, Freedman VA. National Health and Aging Trends Study round 1 user guide: final release. Johns Hopkins University School of Public Health. 2012. Accessed February 1, 2024. https://nhats.org/sites/default/files/2021-01/NHATS_Round_1_User_Guide_Final_Release_0.pdf

[13] Montaquila J, Freedman VA, Kasper JD. National Health and Aging Trends Study round 1 income imputation. NHATS Technical Paper #3. Johns Hopkins University School of Public Health. 2012. Accessed February 1, 2024. https://www.nhats.org/sites/default/files/2021-01/NHATS_Round1_Income_Imputation_11_09_12.pdf

[14] Ornstein KA, Leff B, Covinsky KE, . Epidemiology of the homebound population in the United States. JAMA Intern Med. 2015;175(7):1180-1186. doi:10.1001/jamainternmed.2015.1849 26010119 PMC4749137

[15] Freedman VA, Kasper JD, Spillman BC, . Behavioral adaptation and late-life disability: a new spectrum for assessing public health impacts. Am J Public Health. 2014;104(2):e88-e94. doi:10.2105/AJPH.2013.301687 24328656 PMC3935680

[16] Olaisen RH, Rossen LM, Warner M, Anderson RN. Unintentional injury death rates in rural and urban areas: United States, 1999-2017. NCHS Data Brief. 2019;(343):1-8. 31442193

[17] Bandeen-Roche K, Seplaki CL, Huang J, . Frailty in older adults: a nationally representative profile in the United States. J Gerontol A Biol Sci Med Sci. 2015;70(11):1427-1434. doi:10.1093/gerona/glv133 26297656 PMC4723664

[18] Davydow DS, Zivin K, Langa KM. Hospitalization, depression and dementia in community-dwelling older Americans: findings from the National Health and Aging Trends Study. Gen Hosp Psychiatry. 2014;36(2):135-141. doi:10.1016/j.genhosppsych.2013.11.008 24388630 PMC3951607

[19] Kasper JD, Freedman VA, Spillman B. Classification of persons by dementia status in the National Health and Aging Trends Study. NHATS Technical Paper #5. Johns Hopkins University School of Public Health. 2013. Accessed February 1, 2024. https://www.nhats.org/sites/default/files/inline-files/DementiaTechnicalPaperJuly_2_4_2013_10_23_15.pdf

[20] Addendum for applying for NHATS and NSOC restricted block group files. Accessed October 7, 2024. https://nhats.org/sites/default/files/inline-files/AddendumNHATSNSOCBlockGroup.pdf

[21] About the Neighborhood Atlas: frequently asked questions. Neighborhood Atlas website. Accessed May 1, 2024. https://www.neighborhoodatlas.medicine.wisc.edu

[22] Jarosek S. Death information in the research identifiable Medicare data. Research Data Assistance Center website. Accessed October 8, 2024. https://resdac.org/articles/death-information-research-identifiable-medicare-data

[23] Montaquila J, Freedman VA, Spillman B, Kasper JD. National Health and Aging Trends Study development of round 1 survey weights. NHATS Technical Paper #2. Johns Hopkins University School of Public Health. 2012. Accessed February 1, 2024. https://www.nhats.org/sites/default/files/2021-01/NHATS%20Round%201%20Weighting%20Description_Nov2012_3.pdf

[24] Minimizing disclosure risks when using the NHATS-CMS standard linked files in the NIA Data LINKAGE Program (LINKAGE) enclave. National Health and Aging Trends Study (NHATS). Accessed June 25, 2024. https://www.nhats.org/sites/default/files/2024-03/NHATS%20Restrictions_LINKAGE_Enclave_Standard%20Files%20v2_3_1_2024_2.pdf

[25] Benjamini Y, Hochberg Y. Controlling the false discovery rate: a practical and powerful approach to multiple testing. J R Stat Soc B. 1995;57:289-300.

[26] Lumley T, Lumley MT. Package ‘survey’. Accessed June 13, 2024. http://r.meteo.uni.wroc.pl/web/packages/survey/survey.pdf

[27] Colosimo EA, Ferreira FF, Oliveira MD, Sousa CB. Empirical comparisons between Kaplan-Meier and Nelson-Aalen survival function estimators. J Stat Comput Simul. 2002;72:299-308.

[28] Fay RE. Theory and application of replicate weighting for variance calculations. In: *Proceedings of the Section on Survey Research Methods*. American Statistical Association; 1989:212-219.

[29] Freedman VA, Hu M, DeMatteis J, Kasper JD. Accounting for sample design in NHATS and NSOC analyses: frequently asked questions. NHATS Technical Paper #23, v2. Johns Hopkins University School of Public Health. 2022. Accessed February 1, 2024. https://www.nhats.org/sites/default/files/2021-01/Accounting_for_the_NHATS_NSOC_Design_in_Analyses_FAQ_0.pdf

[30] Hernán MA. The hazards of hazard ratios. Epidemiology. 2010;21(1):13-15. doi:10.1097/EDE.0b013e3181c1ea43 20010207 PMC3653612

[31] Chamberlain AM, St Sauver JL, Finney Rutten LJ, . Associations of neighborhood socioeconomic disadvantage with chronic conditions by age, sex, race, and ethnicity in a population-based cohort. Mayo Clin Proc. 2022;97(1):57-67. doi:10.1016/j.mayocp.2021.09.006 34996566 PMC8775356

[32] Edmonds AT, Rhew IC, Jones-Smith J, . Neighborhood disadvantage, patterns of unhealthy alcohol use, and differential associations by gender, race/ethnicity, and rurality: a study of Veterans Health Administration patients. J Stud Alcohol Drugs. 2022;83(6):867-878. doi:10.15288/jsad.21-00110 36484584 PMC9756400

[33] Montaquila J, Freedman VA, Edwards B, Kasper JD. National Health and Aging Trends Study round 1 sample design and selection. NHATS Technical Paper #1. Johns Hopkins University School of Public Health. 2012. Accessed February 1, 2024. https://www.nhats.org/sites/default/files/2021-01/NHATS%20Round%201%20Sample%20Design%2005_10_12_2.pdf

[34] Buckingham WR, Ryan Powell W, Keller SA, Hansmann KJ, Kind AJH. Bigger isn’t better: why small area geographies are best for actionable index development. Pap Appl Geogr. 2024;10(2):89-95. doi:10.1080/23754931.2024.2312192 39171071 PMC11335328

