# Supplementary material for: Neighborhood Disadvantage in a Nationally Representative Sample of Community-Living Older US Adults
Source: JAMA Netw Open. 2024 Dec 12;7(12):e2450332. doi: 10.1001/jamanetworkopen.2024.50332 (PMC11638794; doi:10.1001/jamanetworkopen.2024.50332)
Supplement: Supplement 1. — eFigure. Census Divisions in the Contiguous United States eTable. Indicators of the Area Deprivation Index at the Census-Block Level [file jamanetwopen-e2450332-s001.pdf]

Supplemental Online Content

Gill TM, Leo-Summers LS, VanderWyk B, Becher RD, Liang J. Neighborhood disadvantage in a nationally representative sample of community-living older adults. *JAMA Netw Open*. 2024;7(12):e2450332. doi:10.1001/jamanetworkopen.2024.50332

eFigure. Census Divisions in the Contiguous United States

eTable. Indicators of the Area Deprivation Index at the Census-Block Level

This supplemental material has been provided by the authors to give readers additional information about their work.

© 2024 Gill TM et al. *JAMA Network Open*

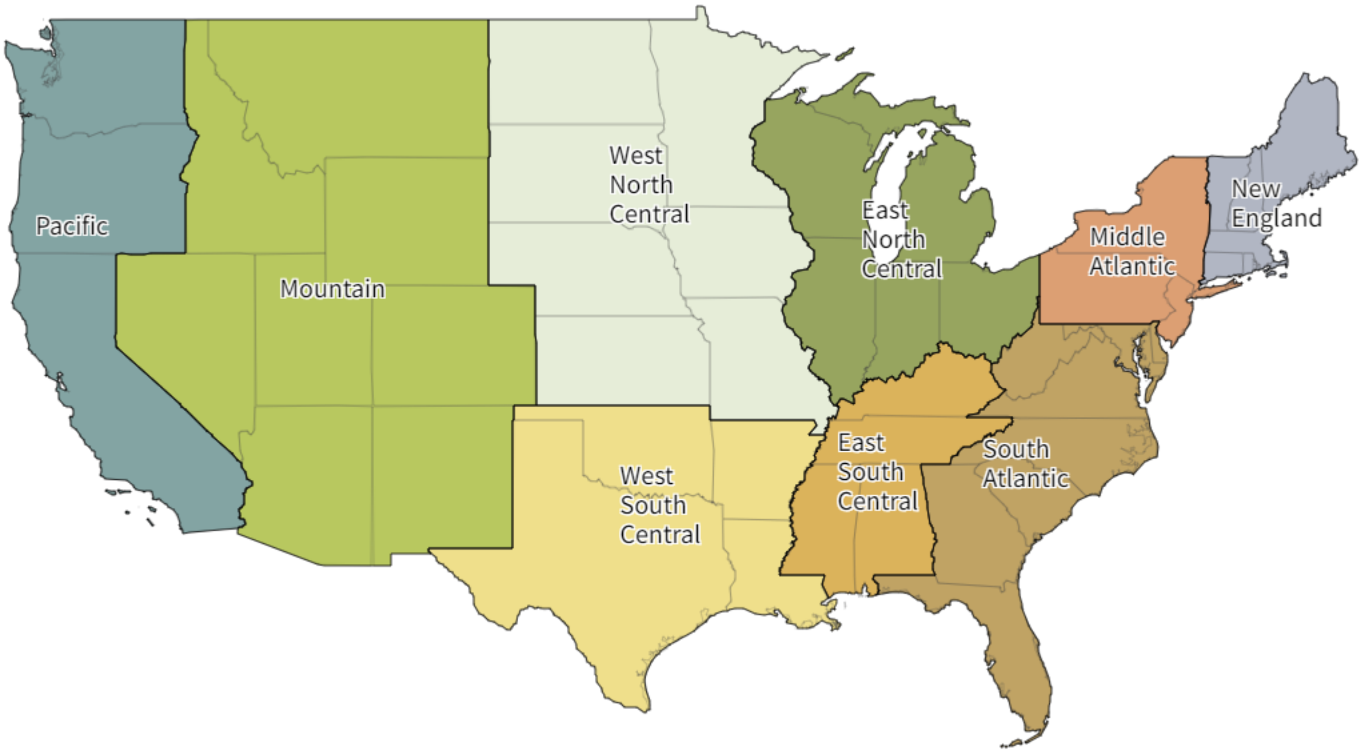

eFigure. Census Divisions in the Contiguous United States

© 2024 Gill TM et al. *JAMA Network Open*

eTable. Indicators of the Area Deprivation Index at the Census-Block Level

1. Percentage of housing units without complete plumbing
2. Percentage population aged ≥25 y with <9 years of education
3. Percentage population aged ≥25 y with <12 years of education
4. Percentage of employed persons aged ≥16 years with white collar occupation
5. Median family income
6. Income disparity
7. Percentage civilian labor force aged ≥16 years
8. Percentage of households with >1 person per room
9. Median home value
10. Median gross rent
11. Median monthly mortgage
12. Percentage occupied housing units
13. Percentage families below the poverty level
14. Percentage population <150% of poverty threshold
15. Percentage of single-parent household with children aged <18 years
16. Percentage of households without motor vehicle
17. Percentage of households without telephone

© 2024 Gill TM et al. *JAMA Network Open*
