# Supplementary material for: Neighborhood Disadvantage in a Nationally Representative Sample of Community-Living Older US Adults
Source: JAMA Netw Open. 2024 Dec 12;7(12):e2450332. doi: 10.1001/jamanetworkopen.2024.50332 (PMC11638794; doi:10.1001/jamanetworkopen.2024.50332)
Supplement: Supplement 2. — Data Sharing Statement [file jamanetwopen-e2450332-s002.pdf]

Data Sharing Statement

Gill. Neighborhood Disadvantage in a Nationally Representative Sample of Community-Living Older US Adults. *JAMA Netw Open*. Published December 12, 2024. doi:10.1001/jamanetworkopen.2024.50332

Data
Data available: No

Additional Information
Explanation for why data not available: The data are restricted by a Data Use Agreement with NHATS.
